# High-risk Return Visits to United States Emergency Departments, 2010–2018

**DOI:** 10.5811/westjem.2022.7.57028

**Published:** 2022-10-18

**Authors:** Dean-An Ling, Chih-Wei Sung, Cheng-Chung Fang, Chia-Hsin Ko, Eric H. Chou, Jeffrey Herrala, Tsung-Chien Lu, Chien-Hua Huang, Chu-Lin Tsai

**Affiliations:** * National Taiwan University Hospital, Department of Emergency Medicine, Taipei, Taiwan; † College of Medicine, National Taiwan University, Department of Emergency Medicine, Taipei, Taiwan; ‡ National Taiwan University Hospital Hsin-Chu Branch, Department of Emergency Medicine, Hsinchu, Taiwan; § Baylor Scott and White All Saints Medical Center, Department of Emergency Medicine, Fort Worth, Texas; ¶ Highland Hospital-Alameda Health System, Department of Emergency Medicine, Oakland, California

## Abstract

**Introduction:**

Although factors related to a return visit to the emergency department (ED) have been reported, only a few studies have examined “high-risk” ED revisits with serious adverse outcomes. In this study we aimed to describe the incidence and trend of high-risk ED revisits in United States EDs and to investigate factors associated with these revisits.

**Methods:**

We obtained data from the National Hospital Ambulatory Medical Care Survey (NHAMCS), 2010–2018. Adult ED revisits within 72 hours of a previous discharge were identified using a mark on the patient record form. We defined high-risk revisits as revisits with serious adverse outcomes, including intensive care unit admissions, emergency surgery, cardiac catheterization, or cardiopulmonary resuscitation (CPR) during the return visit. We performed analyses using descriptive statistics and multivariable logistic regression, accounting for NHAMCS’s complex survey design.

**Results:**

Over the nine-year study period, there were an estimated 37,700,000 revisits, and the proportion of revisits in the entire ED population decreased slightly from 5.1% in 2010 to 4.5% in 2018 (P for trend = 0.02). By contrast, there were an estimated 827,000 high-risk ED revisits, and the proportion of high-risk revisits in the entire ED population remained stable at approximately 0.1%. The mean age of these high-risk revisit patients was 57 years, and 43% were men. Approximately 6% of the patients were intubated, and 13% received CPR. Most of them were hospitalized, and 2% died in the ED. Multivariable analysis showed that older age (65+ years), Hispanic ethnicity, daytime visits, and arrival by ambulance during the revisit were independent predictors of high-risk revisits.

**Conclusion:**

High-risk revisits accounted for a relatively small fraction (0.1%) of ED visits. Over the period of the NHAMCS survey between 2010–2018, this fraction remained stable. We identified factors during the return visit that could be used to label high-risk revisits for timely intervention.

## INTRODUCTION

Unscheduled revisits are often inevitable in the emergency department (ED) and pose a significant burden on patients and clinicians. The causes of an unscheduled revisit could be grouped into several dimensions: they could be associated with patient preference, illnesses, systems of care, and clinicians.^1^ Of these dimensions, patient- or illness-related factors (eg, patients’ preference for treatment venue or natural disease progression) account for most revisits, whereas only 5–10% of unscheduled revisits are associated with suboptimal quality of initial emergency care.^2^–^5^ Given the diverse causes associated with revisits, the use of revisit rate as an indicator of quality has been debated.^6^

For quality assessment, outcomes after revisit have been proposed as alternative quality metrics, including unscheduled ED revisits resulting in hospitalization.^6^ Risk factors for hospitalization after an ED revisit have also been described. Age, illness severity, initial presenting symptoms, and clinician experience have all been associated with hospitalization at revisit.^7^,^8^ Recent studies have examined the subsequent inpatient outcomes to evaluate the validity of this alternative quality metric. Compared with ED patients hospitalized directly at the index visit, those who are hospitalized during ED revisits actually had a lower intensive care unit (ICU) transfer rate and cost during the hospital stay.^9^,^10^ Similar results were also reported among pediatric patients.^11^ One study reported that most hospitalizations after an ED revisit are illness-related.^12^ Taken together, hospitalization after an ED revisit may not imply a care delay or poor quality care during the initial ED visit.

Another promising quality metric would be the incidence and factors associated with high-risk revisits. A small proportion of patients who return to the ED have serious adverse outcomes, such as ICU admission, emergent surgery or intervention, or even cardiac arrest. Studies have highlighted factors that are clinician-related. Timing of the initial ED visit, shorter initial ED management time, presenting symptoms, and certain diagnoses were proposed as likely reasons for revisits with serious adverse outcomes.^13^–^15^ A proportion of these high-risk revisits may hence be avoidable. Most high-risk revisit studies are case series, lacking a comparison group for more robust inferences. Understanding factors associated with high-risk return visits to the ED may help in timely recognition and early interventions to prevent serious adverse events.

Using a US nationally representative sample we aimed to describe the incidence and trends of high-risk return ED visits and to investigate factors associated with these revisits.

## METHODS

### Study Design and Setting

The National Hospital Ambulatory Medical Care Survey (NHAMCS) is a cross-sectional, multistage probability sample of visits to non-institutional general and short-stay hospitals, excluding federal, military, and Veterans Administration hospitals, located in the 50 states and the District of Columbia.^16^ The NHAMCS is conducted annually by the National Center for Health Statistics (NCHS). It covers geographic primary sampling units, hospitals within primary sampling units, EDs within hospitals, and patients within EDs. The number of EDs sampled is approximately 300–400 per year. Trained ED staff collected clinical information during a randomly assigned four-week period for each of the sampled EDs using a structured patient record form (PRF). Data included patient demographics, reason for the visit, diagnosis, procedures, medications given at the visit, and the basic characteristics of the treating physician and hospital. Quality control was performed using a two-way independent verification procedure for a 10% sample of the records. The non-response rate for most items was <5%. The coding error rates were <2%.^17^ Because the NHAMCS contains publicly available, de-identified data, the National Taiwan University Hospital Institutional Review Board exempted this study from review.

**Population Health Research Capsule**

What do we already know about this issue?

*Although factors related to a return visit to the ED have been reported, only a few studies have examined “high-risk” ED revisits with serious adverse outcomes.*

What was the research question?

*We sought to investigate the incidence/trends of high-risk ED revisits and factors associated with these revisits.*

What was the major finding of the study?

*High-risk revisits accounted for 0.1% of the ED visits, and this fraction remained stable between 2010–2018.*

How does this improve population health?

*Albeit rare, catastrophic high-risk revisits should be prevented. We identified factors that could be used to label these revisits for timely intervention.*

### Study Population

We used NHAMCS data from 2010–2018 in this analysis. First, we excluded ED visits made by patients aged <18 years. The PRF contained a revisit variable “seen72,” which indicated whether the patient had been seen in that ED in the prior 72 hours. We further excluded patient visits missing this information. We then divided the study population into the “revisit” and “non-revisit” groups. In the revisit group, we defined high-risk revisits as those by patients with serious adverse outcomes, including ICU admissions, and those who received emergency surgery, cardiac catheterization, or cardiopulmonary resuscitation (CPR) during the return visit.

### Variables

To maintain consistency across years, we recoded race/ethnicity as non-Hispanic White, non-Hispanic Black, Hispanic, and other. Insurance was recoded as private, Medicare, Medicaid or other state-based programs, self-pay, and other. The US regions represented standardized geographical divisions, as defined by the US Census Bureau (Northeast, Midwest, South, and West).^18^ Up to five reasons for each ED visit were coded using the “Reason for Visit Classification for Ambulatory Care,” a standardized sourcebook used in NCHS studies.^19^ We ascertained chronic comorbid conditions based on the PRF, including diabetes mellitus, hypertension, coronary artery disease, and cancer. Data on disease severity/urgency included triage levels, vital signs at triage, and pain scores. Several procedures were documented on the PRF, including CPR and endotracheal intubation. Imaging performed in the ED was also recorded, including computed tomography (CT) and magnetic resonance imaging (MRI). Visit disposition was recorded for each ED visit, including admission to the operating room, cardiac catheterization lab, or ICU. For ED visits resulting in hospitalizations, we recorded inpatient mortality, and hospital length of stay (LOS).

### Outcome Measures

The primary outcome measure was the high-risk revisit rate in the ED, which was calculated as the number of high-risk revisits divided by the total number of adult ED visits. The co-primary outcome measure was the overall ED revisit rate.

### Statistical Analysis

We used Stata 16.0 (StataCorp, College Station, TX) to adjust the variances for the NHAMCS estimates to account for the complex design of the survey. Standard errors (SE) were calculated for the NHAMCS estimates. All statistical tests were based on estimates that had at least 30 cases and a relative SE of <30% (ie, the SE divided by the estimate expressed as a percentage of the estimate) in the sample data, according to the NCHS recommendations. For the high-risk revisit trend analysis, we combined two years of data to increase the stability of the estimates. Descriptive statistics were presented as proportions (with 95% confidence intervals [CI]) or means (with SEs). We used the weighted chi-square test to assess the differences between proportions. Logistic regression models were used to test for significant changes in the primary outcomes (overall and high-risk ED revisit rate) during the study period, in which calendar year was a linear independent variable. We performed multivariable logistic regression analysis to assess the independent predictors of high-risk revisits among overall revisits. Due to the limited number of outcomes, the parsimonious multivariable model included age, gender, race/ethnicity, insurance, season, weekend, time of presentation, geographic region, and arrival mode. Odds ratios (OR) are presented with 95% CI. All *P* values are two-sided, with *P* <0.05 considered statistically significant.

## RESULTS

**Figure 1:**
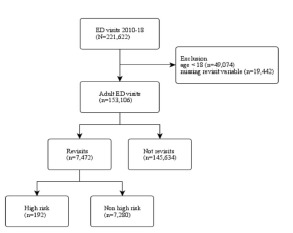
Patient selection process. *ED*, emergency department.

From 2010 to 2018, 221,622 ED visits were recorded in the NHAMCS. After excluding visits from patients aged <18 years (n = 49,074) and missing the revisit variable (n = 19,422), we included a total of 153,106 adult ED visits in the analysis. Of these adult ED visits, 7,472 revisits were within 72 hours, and 145,634 were non-revisits. Of the revisits, 192 were high-risk with serious adverse outcomes. The flowchart is presented in Figure 1. In that same time frame, there were an estimated 842,000,000 adult ED visits. The weighted revisits over the nine-year study period were estimated to be 37,700,000, accounting for 4.5% of the total adult ED population (95% CI 3.9–5.1%). The baseline clinical characteristics of these revisits are summarized in Table 1.

The vast majority of the overall revisit population was aged 18–64 years, predominantly female, and comprised considerable numbers of non-Hispanic Blacks (21%) and Hispanics (15%). Approximately 28% had Medicaid insurance. No particular seasonal variation was noted, and about 40% of the revisits were located in the South. Approximately 18% were sent by ambulance, and 50% were triaged at level 3. Triage vital signs were generally within normal limits. Approximately 16% underwent CT, and very few (0.2~0.3%) had CPR or intubation. The mean ED LOS was about four hours, and 12% were hospitalized. Among those who were hospitalized, 1.2% died during the hospital stay.

**Figure 2:**
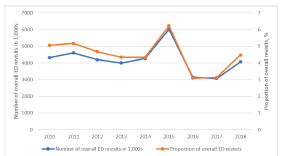
The number and proportion of overall emergency department revisits, 2010–2018. *ED*, emergency department.

Figure 2 depicts the trend in overall ED revisits during the study period. The numbers of overall revisits ranged from 3–6 million with a general decreasing trend. The proportions of revisits among total ED visits decreased slightly from 5.1% in 2010 to 4.5% in 2018 (*P* for trend = 0.02). There were an estimated 827,000 high-risk ED revisits, and the proportion of high-risk revisits within the entire ED population was 0.1% (95% CI 0.07–019%). The baseline clinical characteristics of high-risk revisits are summarized in Table 2.

The elderly aged ≥65 years accounted for 39% of the high-risk revisit population (vs 18% in the overall revisit population). The high-risk revisit population was also predominantly female but comprised a sizable percentage of Hispanics (24%). Approximately 46% had Medicare insurance. The high-risk revisit numbers were higher in the spring, and about 38% of the revisits were located in the South. Approximately 42% were sent by ambulance, and 34% were triaged at levels 1 or 2. The most common presenting symptoms among high-risk revisits included dyspnea (11%), abdominal pain (11%), and chest pain (6%). Triage vital signs showed slightly higher heart rate and respiratory rate, with lower oxygen saturation. Of the high-risk revisit patients, approximately 36% underwent CT, 13% had CPR, and 6% were intubated. The mean ED LOS was about 10 hours, and 88% were hospitalized. Among those who were hospitalized, 6% died during the hospital stay.

**Figure 3:**
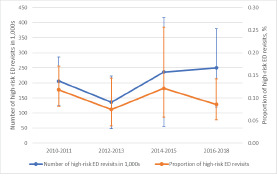
The number and proportion of overall emergency department high-risk revisits, 2010–2018. The error bars represent 95% confidence intervals. ED, emergency department.

Figure 3 depicts the trend in high-risk ED revisits during the study period. The numbers of high-risk revisits ranged from 130,000 to 250,000. The proportions of high-risk revisits among total ED visits remained stable at approximately 0.1% (*P* for trend = 0.86). Of the 37,700,000 weighted revisits, 827,000 (2.2%; 95% CI 1.6–3.1%) were high-risk revisits. Table 3 shows the multivariate analysis of factors associated with high-risk revisits among the overall revisit population during the return visit. Age ≥ 65 years (adjusted odds ratio [aOR] 2.5; 95% CI 1.3–4.8), Hispanic ethnicity (aOR 2.4; 95% CI 1.02–5.4), daytime revisits (aOR 1.5; 95% CI 1.03–2.3), and arrival by ambulance (aOR 3.5; 95% CI 1.7–7.0) were independent predictors of high-risk ED revisits.

## DISCUSSION

Our study showed that of the 842,000,000 adult ED visits represented in the analysis, 37,700,000 (4.5%) were revisits within 72 hours. Of these revisits, 827,000 (2.2%) were high-risk revisits, defined as those with serious adverse outcomes, including being admitted to the ICU or receiving emergency surgery, cardiac catheterization, or CPR. The proportion of high-risk revisits in the entire ED population was 0.1%. During the nine-year study period, high-risk revisit rates remained stable, whereas overall revisits decreased slightly. High-risk revisits had differing characteristics compared to other revisits. Older age, Hispanic ethnicity, daytime revisits, and arrival by ambulance during the ED revisit were associated with serious adverse outcomes.

The overall 72-hour revisit rate of 4.5% in our study is similar to the revisit rates reported in previous studies,^1^ whereas the high-risk revisit rate of 0.1% is higher than the previously reported returned ICU admission rate of approximately 0.05%.^13^–^15^,^20^ This difference is likely attributed to our study’s more comprehensive definition of high-risk revisit, which included both ICU admissions and other serious adverse outcomes. There is a paucity of data regarding US national revisit trends over time for both overall and high-risk revisits. For the first time, this study identified a statistically significant decreasing trend in overall revisits from 2010 to 2018. At the same time, our data suggests that high-risk revisit rates remained stable. These different trends suggest a relatively “fixed” rate of high-risk revisits, as opposed to a relatively “elastic” rate of overall revisits that were multifactorial. Alternatively, the decrease in overall revisits may have resulted from improved ED care, improved referral to primary care following an ED visit, and telehealth applications.^21^,^22^ Further research is required to investigate the persistence of this trend and possible mechanisms associated with the decreased rate of overall revisits observed during this study period.

The characteristics of general ED revisits have been studied, and prediction models to identify general revisits have been developed.^23^–^25^ Prediction models of high-risk revisits are quite limited, as such models would require a large sample size to predict rare events. Prediction can occur at initial ED discharge (most common), between visits, or upon the revisit. We previously employed a case-crossover design to investigate time-varying factors associated with high-risk revisits.^26^ Changes in symptoms to dyspnea or chest pain, changes in arrival mode to ambulance, and changes in certain vital signs were most predictive of severe adverse events on revisits. In the current study, we focused on the prediction on the return visit, ie, identifying high-risk revisits from the pool of general revisits.

We identified several patient and contextual factors associated with serious adverse events on revisits. Elderly patients revisited the ED more frequently^27^,^28^ and were more often admitted after the revisit.^8^,^29^ Our results indicate that elderly patients are also prone to critical events, which is consistent with previous reports.^30^ Frailty, complexity of comorbidities, and declining cognitive and physical function could all contribute to the need for more medical attention.^31^ In addition, we found Hispanic ethnicity to be associated with high-risk revisits compared to overall revisits, in contrast to the findings of fewer rehospitalizations after ED discharge among patients who identified as Hispanic from a previous report.^32^ We hypothesize that language barriers, clinician implicit bias, and inequities in socioeconomic status and access to healthcare resources may have contributed to this disparity. High quality communication is required to properly diagnose and safely disposition patients from the ED. Thus, ongoing efforts should be made to ensure that the future emergency physician workforce reflects its growing Hispanic population in both demographic and linguistic terms ; meanwhile, high quality in-person or tele-interpreters should be readily available within US EDs caring for this patient population.

Regarding the timing of visits, initial ED visits in the evening shifts or during off-hours have been identified as risk factors for subsequent ICU admission on revisits for both adult and pediatric patients.^13^,^15^,^33^ Evidence on the severity of revisits concerning the timing of the return visit is limited. Our study showed that daytime visits were associated with a high-risk revisit, probably because patients deferred medical care until morning. Arriving by ambulance was also linked with a high-risk revisit. Previous studies have reported an association between the mode of transportation and ED admission and ICU admission on revisit.^7^,^30^

## LIMITATIONS

Our study has several limitations. First, the medical records were only retrieved cross-sectionally, and we were unable to trace the revisit to the initial visit. Nevertheless, the data still provided the key characteristics to distinguish high-risk revisits from overall revisits. Second, information on revisits to another healthcare facility was also unavailable, which may have resulted in underestimating the total revisit rate. In our previous study, about one in three ED revisits occurred in another hospital.^34^ Third, we did not include in our study other conditions such as stroke that may also constitute a high-risk revisit. Finally, other factors that may contribute to serious adverse outcomes, such as ED occupancy, number of staff, and seniority of treating clinicians, were not available in the NHAMCS.

## CONCLUSION

We found that high-risk revisits account for approximately 0.1% of adult ED visits in this nationally representative sample. The high-risk revisit rate remained stable during the study period from 2010 to 2018, whereas the overall revisit rate decreased. Older age, Hispanic ethnicity, daytime revisits, and arrival by ambulance are factors associated with high-risk revisits. Much work is needed to reduce these catastrophic adverse events, namely, to develop and validate prediction models at initial ED discharge, between visits, and on return visits. Thus, timely interventions can be implemented on the target populations at different time points for improved quality of care and patient safety.

## Figures and Tables

**Table 1:** Baseline clinical characteristics of emergency department revisit patients, 2010–2018.

Variable	Weighted number or weighted mean	Weighted percentage (95% CI)
Overall	37,700,000	
Age group		
18–64	30,800,000	81.7 (79.9–83.4)
65+	6,884,000	18.3 (16.6–20.1)
Gender		
Male	16,800,000	44.7 (43.1–46.3)
Female	20,800,000	55.3 (53.7–56.9)
Race/ethnicity		
Non-Hispanic White	22,800,000	60.5 (56.2–64.6)
Non-Hispanic Black	7,954,000	21.1 (18.5–23.9)
Hispanic	5,714,000	15.2 (11.0–20.5)
Other	1,226,000	3.3 (1.9–5.5)
Insurance		
Private insurance	8,879,000	26.4 (24.4–28.6)
Medicare	8,925,000	26.6 (24.9–28.4)
Medicaid or state-based programs	9,361,000	27.9 (25.5–30.4)
Self-pay (uninsured)	4,847,000	14.4 (13.0–16.0)
Other	1,576,000	4.7 (3.6–6.1)
Season		
Spring (Mar. – May)	9,726,000	25.8 (21.8–30.2)
Summer (Jun. – Aug.)	9,618,000	25.5 (21.8–29.6)
Fall (Sep. – Nov.)	10,700,000	28.5 (22.0–35.9)
Winter (Dec. – Feb.)	7,614,000	20.2 (16.9–23.9)
Weekend	9,906,000	26.3 (24.9–27.7)
Time of ED presentation		
7:00 AM to 2:59 PM	16,500,000	44.1 (42.2–46.0)
3:00 PM to 10:59 PM	14,700,000	39.3 (37.9–40.8)
11:00 PM to 6:59 AM	6,178,000	16.6 (15.1–18.1)
Geographic region		
Northeast	6,945,000	18.4 (14.5–23.2)
Midwest	7,223,000	19.2 (15.1–24.0)
South	15,000,000	39.7 (31.2–48.9)
West	8,553,000	22.7 (18.2–27.9)
Metropolitan area	28,000,000	83.6 (75.7–89.3)
Arrival by ambulance	6,411,000	17.6 (16.0–19.3)
Number of comorbid conditions*	1.1	1.0–1.2
Most common chief complaints		
Abdominal pain	3,401,000	9.0 (8.0–10.2)
Chest pain	1,494,000	4.0 (3.3–4.8)
Headache	1,226,000	3.3 (2.8–3.8)
Triage level		
1	235,000	0.8 (0.6–1.1)
2	3,276,000	11.0 (9.8–12.4)
3	14,900,000	50.1 (47.5–52.7)
4	9,156,000	30.7 (28.5–33.1)
5	2,188,000	7.3 (6.2–8.8)
Pain score		
Severe (7–10)	14,300,000	49.8 (47.1–52.4)
Moderate (4–6)	5,032,000	17.5 (16.0–19.2)
Mild (1–3)	2,084,000	7.3 (6.3–8.3)
No pain (0)	7,304,000	25.4 (23.6–27.4)
Triage vital signs		
Body temperature, °C	36.8	36.7–36.8
Heart rate, beats per minute	86.3	85.6–87.0
Respiratory rate, breaths per minute	18.9	18.2–19.6
Oxygen saturation, %	97.2	97.0–97.5
Systolic blood pressure, mm Hg	134.9	134.0–135.9
ED management		
Intubation	85,000	0.2 (0.1–0.4)
CPR	108,000	0.3 (0.0–1.7)
Any CT	6,127,000	16.3 (14.7–17.9)
MRI	385,000	1.0 (0.7–1.5)
Length of ED stay, hours	4.1	3.8–4.7
ED disposition		
Admission	4,467,000	11.9 (10.1–13.9)
Died in the ED	58,000	0.2 (0.1–0.4)
Hospitalization		
Length of hospital stay, days	4.8	4.3–5.2
Inpatient mortality	50,000	1.2 (0.6–2.5)

* Available from 2012–2018.

*CI*, confidence interval; *ED*, emergency department; *CPR*, cardiopulmonary resuscitation; *mm Hg*, millimeters of mercury; *CT*, computed tomography. *MRI*, magnetic resonance imaging.

**Table 2:** Baseline clinical characteristics of emergency department high-risk revisit patients, 2010–2018.

Variable	Weighted number or weighted mean	Weighted percentage (95% CI)
Overall	827,000	
Age group		
18–64	507,000	61.2 (49.9–71.4)
65+	321,000	38.8 (28.6–50.1)
Gender		
Male	357,000	43.2 (33.5–53.3)
Female	470,000	56.8 (46.7–66.5)
Race/ethnicity		
Non-Hispanic White	478,000	57.7 (40.3–73.4)
Non-Hispanic Black	126,000	15.2 (8.8–24.9)
Hispanic	201,000	24.3 (9.1–50.1)
Other	22,000	2.7 (1.1–6.7)
Insurance		
Private insurance	158,000	22.0 (14.3–32.2)
Medicare	329,000	45.7 (35.7–56.1)
Medicaid or state-based programs	117,000	16.3 (9.4–26.6)
Self-pay (uninsured)	51,000	7.2 (3.4–14.3)
Other	63,000	8.8 (4.2–17.7)
Season		
Spring (Mar. – May)	281,000	33.9 (22.5–47.6)
Summer (Jun. – Aug.)	191,000	23.1 (15.2–33.5)
Fall (Sep. – Nov.)	193,000	23.3 (13.9–36.4)
Winter (Dec. – Feb.)	163,000	19.7 (14.1–26.8)
Weekend	200,000	24.1 (15.6–35.3)
Time of ED presentation		
7:00 AM to 2:59 PM	408,000	50.0 (40.0–60.0)
3:00 PM to 10:59 PM	250,000	30.6 (22.5–40.1)
11:00 PM to 6:59 AM	158,000	19.4 (12.4–28.9)
Geographic region		
Northeast	132,000	16.0 (9.0–26.9)
Midwest	134,000	16.2 (8.7–28.1)
South	317,000	38.3 (21.1–58.9)
West	245,000	29.6 (17.5–45.3)
Metropolitan area	747,000	93.5 (84.5–97.5)
Arrival by ambulance	330,000	42.3 (29.1–56.7)
Number of comorbid conditions*	2.1	1.6–2.5
Most common chief complaints		
Shortness of breath	93,000	11.3 (6.5–18.9)
Abdominal pain	92,000	11.2 (4.7–24.3)
Chest pain	48,000	5.8 (2.7–12.2)
Triage level		
1	51,000	8.9 (4.6–16.5)
2	144,000	25.0 (17.1–35.2)
3	332,000	57.7 (47.6–67.2)
4	37,000	6.4 (3.6–10.9)
5	12,000	2.0 (0.5–7.3)
Pain score		
Severe (7–10)	212,000	43.1 (32.2–54.8)
Moderate (4–6)	83,000	16.8 (9.2–28.7)
Mild (1–3)	21,000	4.3 (1.6–11.1)
No pain (0)	176,000	35.8 (25.2–47.9)
Triage vital signs		
Body temperature, °C	36.8	36.6–36.9
Heart rate, beats per minute	91.1	86.0–96.2
Respiratory rate, breaths per minute	20.7	19.0–22.4
Oxygen saturation, %	95.2	93.6–96.7
Systolic blood pressure, mm Hg	134.2	128.6–139.8
ED management		
Intubation	48,000	5.8 (2.8–11.5)
CPR	108,000	13.1 (2.5–46.7)
Any CT	300,000	36.2 (25.4–48.7)
MRI	59,000	7.1 (2.0–22.6)
Length of ED stay, hours	9.5	3.3–15.8
ED disposition		
Admission	728,000	88.0 (55.3–97.8)
Died in the ED	19,000	2.3 (0.6–8.9)
Hospitalization		
Length of hospital stay, days	4.5	3.8–5.2
Inpatient mortality	44,000	6.4 (2.8–13.6)

* Available from 2012–2018.

*CI*, confidence interval; *ED*, emergency department; mm Hg, millimeters of mercury; *CPR*, cardiopulmonary resuscitation; *CT*, computed tomography; *MRI*, magnetic resonance imaging.

**Table 3:** Emergency department visit rates for high-risk revisit, overall, stratified, and multivariable analysis, 2010–2018.

Variable	Proportion of high-risk revisit, % (95% CI)	Adjusted OR (95% CI)*
Overall	**1.4**	
Age group, years		
18–64	1.6 (1.2–2.3)	1.0 (reference)
65+	4.7 (2.8–7.6)	**2.5 (1.3–4.8)**
Gender		
Male	2.1 (1.4–3.3)	0.9 (0.6–1.3)
Female	2.3 (1.6–3.2)	1.0 (reference)
Race/ethnicity		
Non-Hispanic White	2.1 (1.5–2.9)	1.0 (reference)
Non-Hispanic Black	1.6 (1.0–2.6)	0.9 (0.5–1.7)
Hispanic	3.5 (1.5–8.2)	**2.4 (1.02–5.4)**
Other	1.8 (0.6–5.1)	1.3 (0.4–4.5)
Insurance		
Private insurance	1.8 (1.1–2.8)	1.0 (reference)
Medicare	3.7 (2.3–5.9)	0.9 (0.4–2.0)
Medicaid or state-based programs	1.2 (0.7–2.1)	0.7 (0.3–1.3)
Self-pay (uninsured)	1.1 (0.5–2.4)	0.6 (0.2–1.4)
Other	4.0 (1.8–8.7)	1.7 (0.9–2.9)
Season		
Spring (Mar. – May)	2.9 (1.6–5.2)	1.4 (0.8–2.7)
Summer (Jun. – Aug.)	2.0 (1.4–2.9)	1.0 (reference)
Fall (Sep. – Nov.)	1.8 (1.0–3.1)	0.9 (0.4–1.8)
Winter (Dec. – Feb.)	2.1 (1.4–3.4)	1.1 (0.6–1.9)
Weekend		
Non-weekend	2.3 (1.5–3.3)	1.0 (reference)
Weekend	2.0 (1.2–3.3)	1.0 (0.5–1.8)
Time of ED presentation		
7:00 AM to 2:59 PM	2.5 (1.7–3.6)	**1.5 (1.03–2.3)**
3:00 PM to 10:59 PM	1.7 (1.1–2.6)	1.0 (reference)
11:00 PM to 6:59 AM	2.6 (1.4–4.7)	1.2 (0.6–2.4)
Geographic region		
Northeast	1.9 (1.2–3.1)	1.0 (reference)
Midwest	1.9 (1.1–3.2)	1.2 (0.5–2.8)
South	2.1 (1.0–4.5)	1.4 (0.6–3.0)
West	2.9 (1.8–4.5)	1.1 (0.5–2.3)
Arrival mode		
Arrival not by ambulance	1.5 (0.9–2.6)	1.0 (reference)
Arrival by ambulance	5.1 (3.8–6.9)	**3.5 (1.7–7.0)**

* Multivariable model adjusts for all variables in the table.

Significant odds ratios are highlighted in bold.

*OR*, odds ratio; *CI*, confidence interval; *ED*, emergency department.
